# Supplementary material for: Trends and Patterns in Electronic Health Record Research (1991–2022): A Bibliometric Analysis of Australian Literature
Source: Int J Environ Res Public Health. 2024 Mar 19;21(3):361. doi: 10.3390/ijerph21030361 (PMC10970652; doi:10.3390/ijerph21030361)

Figure S1: Identification and selection of articles for bibliometric analysis

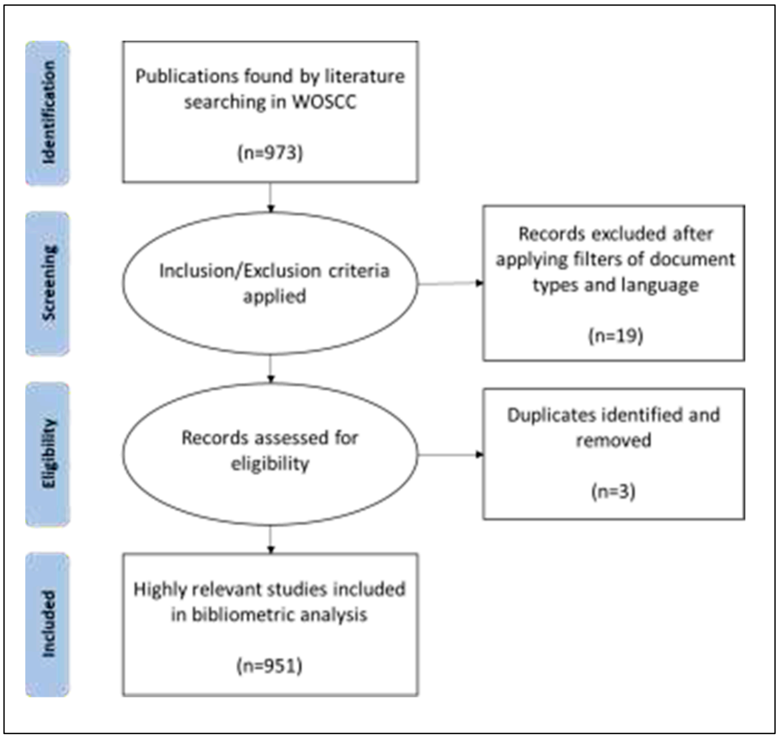

Figure S2: Most relevant authors, affiliations, sources and locally cited sources

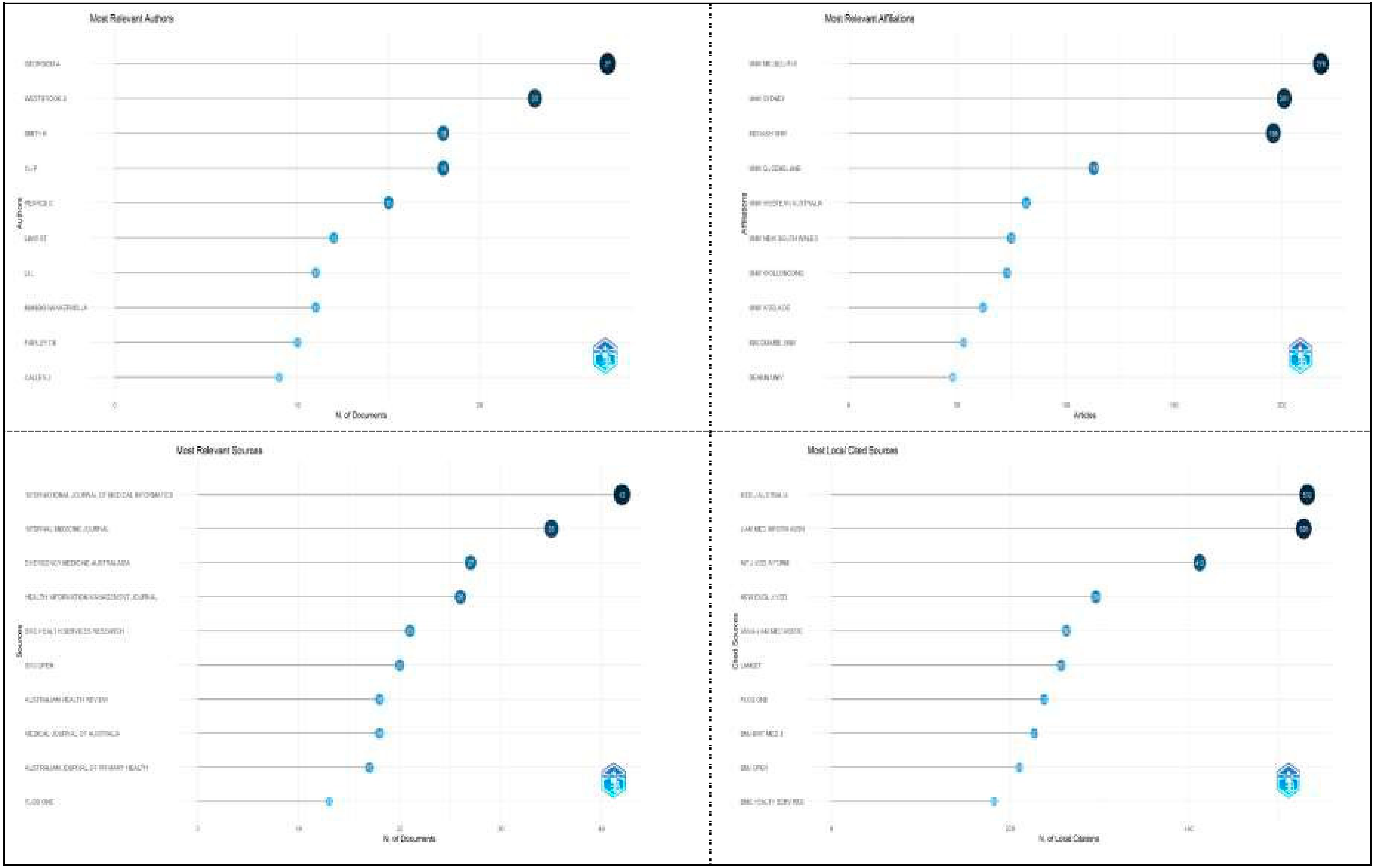

Figure S3: Tree map on most used words on EHR research in Australia

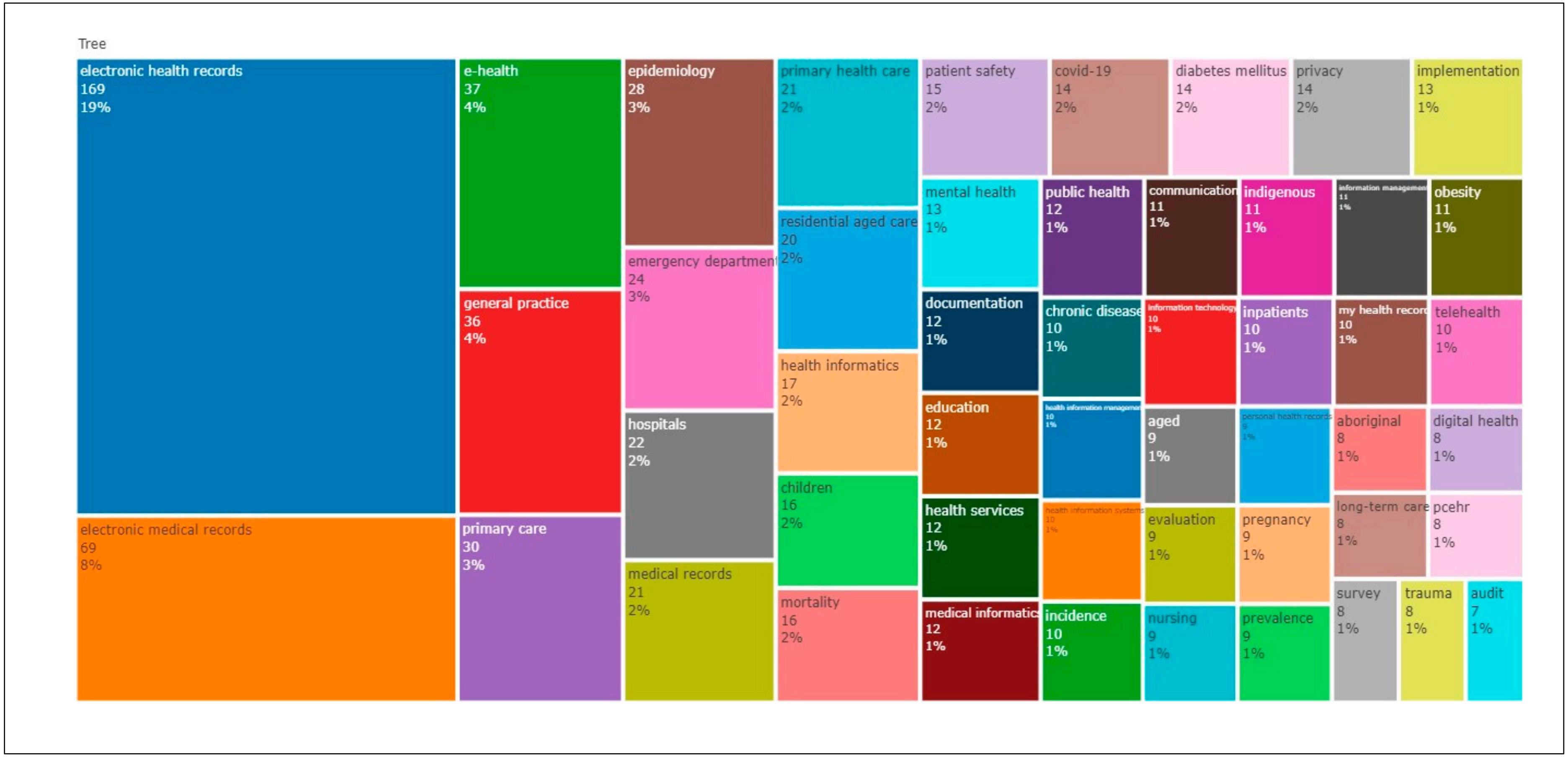

Supplement: Supplementary file 1 [file ijerph-21-00361-s001.zip › ijerph-2840003-supplementary.pdf]
